# Malignancy Risk Within Hyperfunctioning Thyroid Nodules: Clinicopathologic Features and Diagnostic Implications

**DOI:** 10.3390/diagnostics16142249

**Published:** 2026-07-18

**Authors:** Pang-Jui Huang, Shun-Yu Chi, Shun-Chen Huang, Yen-Hsiang Chang, Chen-Kai Chou

**Affiliations:** 1 Division of Endocrinology and Metabolism, Department of Internal Medicine, Kaohsiung Chang Gung Memorial Hospital, Kaohsiung 833, Taiwan; mpj035@cgmh.org.tw; 2 Departments of Surgery, Kaohsiung Chang Gung Memorial Hospital, Kaohsiung 833, Taiwan; abraban@cgmh.org.tw; 3 Department of Anatomic Pathology, Kaohsiung Chang Gung Memorial Hospital, Chang Gung University, Kaohsiung 833, Taiwan; shuang@cgmh.org.tw; 4 Department of Nuclear Medicine, Kaohsiung Chang Gung Memorial Hospital, Kaohsiung 833, Taiwan; changyh@cgmh.org.tw; 5 College of Medicine, Chang Gung University, Kaohsiung 833, Taiwan

**Keywords:** hyperfunctioning neoplasm, hyperthyroidism, radioiodine, technetium-99m, thyroid neoplasms, thyroid nodule

## Abstract

**Background/Objectives**: Hyperfunctioning thyroid nodules are generally considered to have a low malignancy risk, but data on genuine hyperfunctioning thyroid neoplasms remain limited. This study aimed to characterize the clinicopathologic features and diagnostic implications of true hyperfunctioning thyroid neoplasms through lesion-level imaging-pathologic correlation and to estimate their observed frequency within a surgically treated cohort. **Methods**: We retrospectively reviewed 82 hyperthyroid patients at our institution with thyroid scintigraphy suggestive of solitary or multiple toxic nodules between 2014 and 2024; 25 underwent thyroidectomy. True hyperfunctioning thyroid neoplasms were defined by lesion-level imaging-pathologic correlation. **Results**: Among the 25 surgically treated patients, seven had confirmed hyperfunctioning carcinoma or tumors of uncertain malignant potential (UMP) (28.0%; 95% CI, 14.1–47.8%). When referenced against the full scintigraphic cohort of 82 patients, of whom only 30.5% underwent surgery, the observed rate was 8.5% (95% CI, 4.2–16.6%); however, this figure should be interpreted with caution given the inherent surgical selection bias. Four patients had overt carcinomas. The remaining three patients had UMPs. In nodule-based analysis, hyperfunctioning neoplasms were significantly larger than coexisting tumors (5.0 ± 2.8 vs. 0.8 ± 0.7 cm, *p* = 0.007) and more often follicular-patterned (85.7% vs. 27.3%; RR, 3.14; 95% CI, 1.14 to 8.64; *p* = 0.0498). **Conclusions**: These findings indicate that hyperfunctioning status does not definitively exclude malignancy, while also emphasizing that the absolute malignancy frequency in unselected hyperthyroid populations remains uncertain.

## 1. Introduction

Hyperthyroidism caused by autonomously functioning thyroid nodules or toxic multinodular goiter is a common clinical scenario in thyroid practice. In patients with a thyroid nodule and suppressed thyrotropin, current guidelines recommend radionuclide scintigraphy to determine whether the nodule is hyperfunctioning, and a definitively hyperfunctioning nodule is generally considered to have a low pretest probability of malignancy. Accordingly, routine fine-needle aspiration (FNA) of a clearly hyperfunctioning nodule is usually not recommended in standard thyroid nodule algorithms [1,2,3,4].

In real-world practice, evaluation of nodular hyperthyroidism is not based on scintigraphy alone. High-resolution ultrasonography frequently detects additional nodules, bilateral nodular disease, or suspicious sonographic features that influence subsequent management. Recent data also suggest that autonomously functioning thyroid nodules often fall into intermediate-risk ultrasound categories and may yield indeterminate cytology when aspirated, highlighting the practical overlap between functional and sonographic risk stratification [5,6]. Under these circumstances, some hyperthyroid patients still undergo FNA and surgery according to general nodule-management pathways rather than functional status alone.

Although hyperfunctioning nodules have long been regarded as largely benign, this assumption has been questioned by several case series and reviews. Carcinoma arising within hyperfunctioning nodules has been documented in case reports and summarized in subsequent reviews, indicating that hot nodules are not invariably benign [7,8,9,10]. Prior reviews further indicate that these tumors tend to be relatively large (mean size > 4 cm), and follicular thyroid carcinoma accounting for approximately 36–47% of reported primary cases [9,10]. Nevertheless, the overall odds of malignancy remain lower than in non-toxic nodules [11]. Furthermore, contemporary pathology recognizes that this functional neoplastic spectrum extends beyond overt carcinoma to include borderline entities, such as follicular tumors of uncertain malignant potential (FT-UMP), further complicating diagnostic and surgical decision-making [12,13].

A related challenge in interpreting the literature is lesion attribution. In hyperthyroid glands with multiple nodules, reported malignancies may arise either from the hyperfunctioning nodule itself or from separate coexisting nonfunctioning nodules. Lee et al. demonstrated that thyroid cancers can occur in both locations and emphasized that ultrasonography is required to detect coexisting malignant nodules that may be missed if attention is focused only on the hot nodule [14]. Previous Taiwanese surgical series have reported concurrent thyroid cancer in hyperthyroid patients, including toxic nodular disease; however, these studies did not determine whether the tumor arose from the hyperfunctioning nodule itself or from a separate coexisting tumor [15,16,17].

In this study, we retrospectively reviewed hyperthyroid patients with scintigraphic findings suggestive of toxic nodular disease to characterize the clinicopathologic features and diagnostic implications of true hyperfunctioning thyroid neoplasms through lesion-level functional-pathologic correlation and to estimate their observed frequency within a surgically treated cohort. By incorporating lesion-level functional-pathologic correlation, we aimed to distinguish verified hyperfunctioning thyroid neoplasms from coexisting malignancies and to better define their clinicopathologic significance.

## 2. Materials and Methods

This study was approved by the Institutional Review Board of Kaohsiung Chang Gung Memorial Hospital.

### 2.1. Study Population

**Figure 1:**
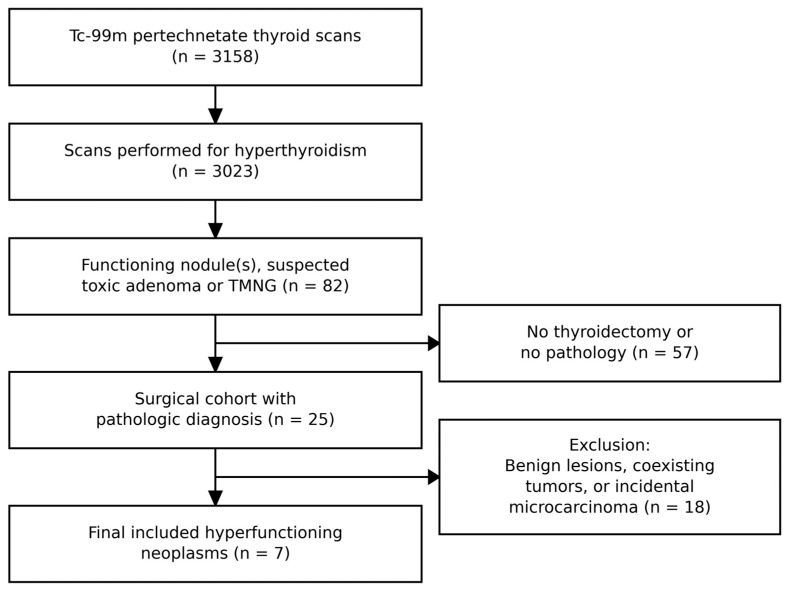
Flow diagram of patient selection and identification of hyperfunctioning thyroid neoplasms.

We reviewed the medical records of patients who underwent thyroid scintigraphy for evaluation of biochemically confirmed or clinically suspected hyperthyroidism at Kaohsiung Chang Gung Memorial Hospital between April 2014 and June 2024. Patients with scintigraphic findings suggestive of functioning thyroid nodule(s), including autonomously functioning thyroid nodule (AFTN) or toxic multinodular goiter (TMNG), were identified for cohort assembly. Among these patients, only those who subsequently underwent thyroid surgery with available histopathological diagnoses were included in the surgical cohort. (Figure 1).

To accurately identify true hyperfunctioning neoplasms, cases within the surgical cohort were carefully evaluated. Cases were not classified as hyperfunctioning neoplasms if the resected tumor arose from a non-hyperfunctioning nodule distinct from the hyperfunctioning focus, arose from the contralateral lobe, or if imaging–pathology correlation was indeterminate. Additionally, incidental papillary thyroid microcarcinomas (≤1 cm) were excluded from this target group, as their functional correlation with scintigraphic uptake cannot be reliably established at this spatial resolution. Cases fulfilling these criteria were classified as hyperfunctioning thyroid neoplasms for the final lesion-level analysis, including overt carcinoma and neoplasms of uncertain malignant potential.

### 2.2. Imaging

Thyroid scintigraphy was primarily performed using technetium-99m (Tc-99m) pertechnetate following standard institutional protocols. In a selected subset of cases requiring further clarification of functional autonomy, iodine-131 (I-131) scintigraphy was additionally performed (annotated for the final target cases in Supplementary Table S1). A hyperfunctioning nodule was defined as a discrete focus of increased radionuclide uptake with relative suppression of surrounding thyroid tissue. Scintigraphic patterns were categorized based on the number of hyperfunctioning foci: a solitary hyperfunctioning focus was classified as AFTN, whereas the presence of multiple hyperfunctioning nodules was classified as TMNG [18].

### 2.3. Data Collection

Within our retrospective study, preoperative thyroid ultrasonography and fine-needle aspiration (FNA) were performed as part of routine clinical care. Clinicopathologic data collected from medical records included patient demographics, thyroid function tests, thyroid autoantibodies, scintigraphic patterns, preoperative ultrasound features, and preoperative FNA results (reported using the Bethesda System for Reporting Thyroid Cytopathology). Biochemical data were extracted from the medical record at the time of hyperthyroidism diagnosis or, when unavailable, from the nearest available laboratory measurements obtained before or around the time of thyroid scintigraphy. Preoperative ultrasound findings were abstracted from available imaging records and categorized according to the American Thyroid Association (ATA) sonographic pattern system. Details regarding the extent of surgical management and final histopathologic diagnoses were also extracted. Surgical specimens were evaluated by experienced endocrine pathologists and classified according to the WHO Classification of Endocrine Tumors.

To ensure consistency, the maximum tumor dimension was primarily determined based on precise microscopic measurements from the final histopathologic report. For exceptionally large tumors with indistinct microscopic boundaries, gross size documented in the pathology report was adopted. In instances where pathological size measurements were indeterminate, the maximum diameter measured on preoperative ultrasound or cross-sectional imaging (e.g., computed tomography) was utilized as a surrogate.

### 2.4. Statistical Analysis

Descriptive statistics were presented as number (percentage), mean ± standard deviation, or median (interquartile range), as appropriate to summarize baseline characteristics. Between-group comparisons were performed using Student’s *t*-test or Welch’s unequal-variance *t*-test for normally distributed continuous variables, and the Mann–Whitney U test for non-normally distributed variables. Categorical variables were compared using the chi-square test or Fisher’s exact test. Statistical analyses were performed using IBM SPSS Statistics (version 26), and a two-sided *p*-value < 0.05 was considered statistically significant.

## 3. Results

### 3.1. Source Population and Scintigraphic Patterns

During the study period between April 2014 and June 2024, 3158 Tc-99m pertechnetate thyroid scans were performed, of which 3023 were obtained for evaluation of hyperthyroidism. Among these, 82 patients were identified as having functioning thyroid nodule(s) with scintigraphic findings suggestive of toxic adenoma or toxic multinodular goiter. The mean age of the overall cohort was 58.5 years (range, 24–88 years), with a marked female predominance (63 females, 76.8%). Thyroid scintigraphy demonstrated substantial functional heterogeneity: a solitary autonomously functioning thyroid nodule (AFTN) was the most frequent trapping pattern (45/82, 54.9%), whereas a multinodular hyperfunctioning pattern consistent with TMNG was observed in 37/82 (45.1%). Baseline biochemical profiles are summarized in Table 1, including suppressed TSH (median 0.010 μIU/mL) and elevated thyroid hormone levels (free T4 and T3). The absence of significant TSH suppression in a few cases was likely related to the administration of antithyroid drugs (ATDs) before the thyroid scan. When comparing AFTN and TMNG subgroups (Table 1), age and thyroid function parameters were similar between groups (all *p* > 0.05). TMNG showed a higher proportion of females than AFTN (89.2% vs. 66.7%, *p* = 0.032). Surgery was performed in 17/45 (37.8%) patients with AFTN and 8/37 (21.6%) patients with TMNG (*p* = 0.150).

### 3.2. Surgical Cohort Classification and Patient-Level Comparison

Of these 82 patients, 25 (30.5%) subsequently underwent thyroidectomy and constituted the surgical cohort for lesion-level pathologic correlation. Postoperative histopathology identified overall carcinoma or UMP in 15 patients (60.0%), whereas the remaining 10 (40.0%) had benign pathology, including follicular adenoma or nodular goiter. After application of the study definition for true hyperfunctioning neoplasms, seven patients were ultimately classified as having confirmed hyperfunctioning thyroid carcinoma or UMP. Of these seven patients, four had overt carcinomas and three had UMPs. On pathological re-review, all three UMPs showed focal equivocal capsular invasion, which was insufficient for a diagnosis of carcinoma. The remaining eight patients with coexisting malignancies were excluded from the clinical comparison in Table 2, but were retained for the subsequent nodule-based analysis. Within the overall cohort (Table 1), hyperfunctioning carcinoma/UMP was more frequently identified in the AFTN subgroup than in the TMNG subgroup (13.3% vs. 2.7%), although this difference did not reach statistical significance (*p* = 0.121).

Compared with the benign pathology group within the surgical cohort (Table 2), the hyperfunctioning carcinoma or tumors of uncertain malignant potential (CA/UMP) group showed no statistically significant differences in age, sex, TSH, free T4, scan impression, extent of surgery, sonographic pattern, or preoperative FNA categories, although indeterminate cytology (Bethesda III/IV) was numerically more frequent in the hyperfunctioning CA/UMP group (57.1% vs. 20.0%).

### 3.3. Clinicopathologic Characteristics of Hyperfunctioning Thyroid Carcinoma/UMP

Among the 25 surgically treated patients, seven cases were confirmed as hyperfunctioning thyroid carcinoma/UMP, representing 28.0% (95% CI, 14.1 to 47.8%) of surgically treated patients. When referenced against the full scintigraphic cohort of 82 patients, of whom only 30.5% underwent surgery, the observed rate was 8.5% (95% CI, 4.2 to 16.6%); however, this figure should be interpreted with caution given the inherent surgical selection bias. Two underwent lobectomy and five underwent total thyroidectomy. The cohort included five females and two males, with mean age 47.3 ± 17.6 years (range, 24 to 68). The mean maximal tumor size was 5.0 ± 2.8 cm, and four cases (57.1%) measured >4 cm. Histologically, follicular-patterned neoplasms predominated. Of the seven cases, FT-UMP was identified in 2 cases (28.6%), FTC in 1 (14.3%), FV-PTC in 1 (14.3%), and classic PTC in 1 (14.3%). The remaining two cases (28.6%) were mixed/collision tumors, consisting of a hyperfunctioning FTC or oncocytic (Hürthle cell) thyroid tumor of uncertain malignant potential (FT-UMP and OT-UMP, respectively), each with coexisting papillary thyroid microcarcinoma. Thus, four of the seven lesion-verified hyperfunctioning neoplasms were carcinomas; three lesions were borderline neoplasms, including two follicular tumors of uncertain malignant potential and one oncocytic tumor of uncertain malignant potential. Accordingly, overt carcinomas and UMPs accounted for 16.0% and 12.0% of the surgically treated cohort, respectively. The papillary thyroid microcarcinomas identified in the two mixed/collision cases were classified as separate coexisting incidental tumors and were not included in the malignant-potential classification of the hyperfunctioning lesion. Detailed clinicopathologic features are summarized in Supplementary Table S1. All seven patients with hyperfunctioning thyroid carcinoma/UMP presented with overt hyperthyroidism and suppressed TSH levels at diagnosis. Thyroid-stimulating hormone receptor antibodies (TSHRAb) were negative, and thyroid peroxidase antibodies (TPOAb) were undetectable in all seven cases, making concomitant autoimmune Graves’ disease less likely as the primary cause of hyperthyroidism in these cases.

**Figure 2:**
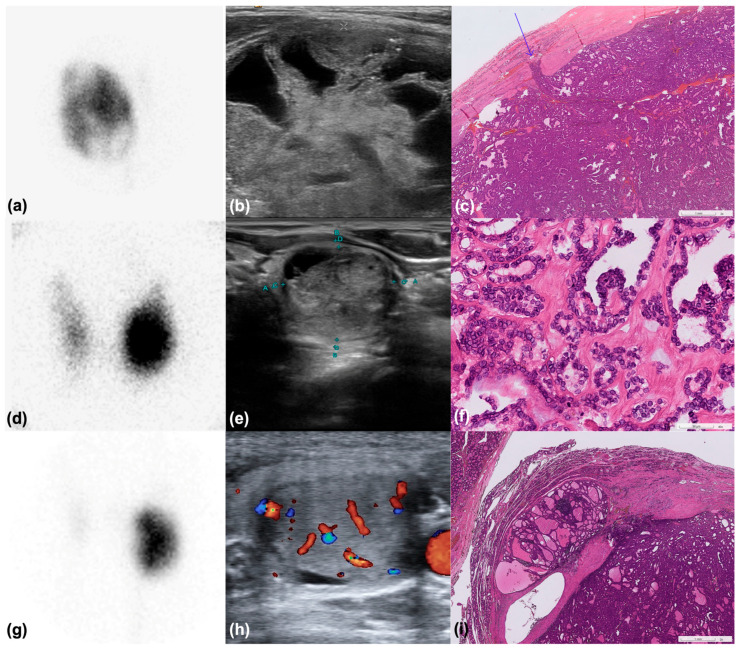
Representative hyperfunctioning thyroid neoplasms with imaging–pathologic correlation in Patient No. 1 (24-year-old female, FTC, 5.5 cm), Patient No. 7 (30-year-old female, PTC, 1.3 cm), and Patient No. 6 (37-year-old female, FT-UMP, 3.5 cm). (**a**–**c**) Thyroid Tc-99m scintigraphy, ultrasonography, and histopathology (H&E, 2×) of Patient No. 1 show a hyperfunctioning right thyroid toxic adenoma, the corresponding dominant nodule with cystic change, and follicular carcinoma with capsular minimal invasion (blue arrow). (**d**–**f**) I-131 thyroid scintigraphy, ultrasonography, and histopathology (H&E, 40×) of Patient No. 7 demonstrate a hyperfunctioning left thyroid toxic adenoma, the corresponding mixed isoechoic nodule, and typical nuclear features of papillary thyroid carcinoma. (**g**–**i**) I-131 thyroid scintigraphy, ultrasonography, and histopathology (H&E, 2×) of Patient No. 6 show a hyperfunctioning left thyroid toxic adenoma, the corresponding nodule, and an encapsulated follicular-patterned tumor with indeterminate capsular extension.

On ultrasound, these hyperfunctioning neoplasms were most commonly solid or mixed-echoic nodules with well-defined margins and generally lacked classic high-risk sonographic features such as marked hypoechogenicity, irregular margins, or suspicious calcifications. Preoperative FNA cytology frequently yielded non-diagnostic or indeterminate results; one case was interpreted as benign (Bethesda II) but was malignant on final pathology (FV-PTC). In the case of papillary thyroid carcinoma arising from a hyperfunctioning nodule, immunohistochemical analysis demonstrated negative BRAF V600E staining, with no evidence of ALK or NTRK overexpression, and a low Ki-67 labeling index, supporting the absence of high-grade or dedifferentiated features. Regional lymph node status was not routinely assessed, as prophylactic lymph node dissection was not performed due to the absence of preoperative suspicion for lymph node metastasis. The mean postoperative follow-up duration was approximately 50 months, during which no structural recurrence or distant metastasis was observed in the hyperfunctioning carcinoma/UMP group. Figure 2 shows representative cases of hyperfunctioning thyroid neoplasms.

### 3.4. Nodule-Level Comparison of Hyperfunctioning and Coexisting Neoplasms

**Figure 3:**
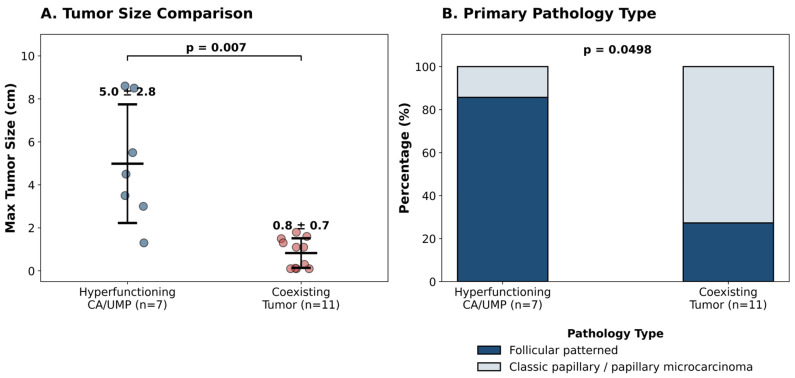
Nodule-based comparison of malignant/UMP nodules: hyperfunctioning vs. coexisting tumors. (**A**) Tumor size. (**B**) Primary Pathology type.

To further delineate lesion-level differences, a nodule-based analysis was performed among the 18 malignant or UMP nodules identified in surgically treated patients. Of these, seven nodules represented hyperfunctioning carcinoma/UMP corresponding to the scintigraphic hot focus, whereas 11 nodules represented coexisting tumors arising in separate nodules, as shown in Figure 3 and detailed in Supplementary Table S2. Specifically, 9 of these coexisting nodules originated from the 8 patients excluded from the earlier patient-level analysis, while the remaining 2 nodules were found concurrently in patients who also harbored a definitive hyperfunctioning neoplasm.

A marked and statistically significant difference in tumor size was observed. Hyperfunctioning carcinoma/UMP nodules demonstrated a mean maximal diameter of 5.0 ± 2.8 cm compared with 0.8 ± 0.7 cm in coexisting tumors (*p* = 0.007, Welch’s *t*-test). Notably, coexisting tumors were predominantly microcarcinomas, whereas hyperfunctioning carcinoma/UMP nodules represented clinically dominant masses. Pathologically, hyperfunctioning nodules were more likely than coexisting tumors to exhibit follicular-patterned histology (85.7% vs. 27.3%; RR, 3.14; 95% CI, 1.14–8.64; *p* = 0.0498), whereas coexisting tumors were primarily classic papillary thyroid carcinoma or papillary microcarcinoma. Furthermore, a sensitivity analysis excluding incidental microcarcinomas (e.g., PTMC, <1 cm) was performed (Supplementary Table S3). The size difference between hyperfunctioning CA/UMP nodules and coexisting clinically significant tumors (≥1 cm) remained statistically significant (5.0 ± 2.8 cm vs. 1.4 ± 0.3 cm, *p* = 0.014). In contrast, the difference in pathology distribution was no longer statistically significant after exclusion of microcarcinoma (*p* = 0.266).

### 3.5. Clinical and Sonographic Follow-Up of the Non-Surgical Cohort

To provide clinical context for the patients without pathologic confirmation, available post-index follow-up data were reviewed for the 57 patients with functional nodules who did not undergo thyroid surgery. Clinical follow-up was available for 41 patients (71.9%), with a mean follow-up duration of 3.9 years. Repeat thyroid ultrasonography was available for 25 patients. Some macrocalcifications identified on the initial ultrasonography persisted on follow-up imaging and were associated with posterior acoustic shadowing. No newly documented high-suspicion sonographic features were identified in the available follow-up ultrasound reports. Subsequent FNA was performed in 17 patients and yielded Bethesda II cytology in 13 patients and Bethesda I cytology in 4 patients; no malignant cytology was documented. No patient with available clinical follow-up was clinically diagnosed with thyroid malignancy during the follow-up period.

## 4. Discussion

Our study evaluated the clinicopathologic features of thyroid neoplasms in hyperthyroid patients by correlating preoperative functional scintigraphy with postoperative surgical pathology on a lesion-level basis. In our cohort, neoplasms arising from the hyperfunctioning nodule were substantially larger than tumors identified in separate coexisting nodules and were predominantly follicular-patterned, whereas coexisting tumors were more often small classic papillary thyroid carcinomas or papillary thyroid microcarcinomas. These findings are broadly consistent with prior reviews of hyperfunctioning thyroid carcinoma [9,10], while also underscoring the distinct clinicopathologic profile of coexisting tumors identified in the same setting.

In the surgical cohort, hyperfunctioning neoplasms and the benign group did not differ significantly in baseline demographic characteristics, thyroid function parameters, or scintigraphic pattern, suggesting that these neoplasms may lack distinct preoperative clinical or biochemical hallmarks. Reported thyroid cancer prevalence in hyperthyroidism has varied widely across studies, likely reflecting differences in patient selection, surgical indication, and endpoint definition [15,16,17,19]. The observed rate of verified hyperfunctioning carcinoma or UMP in our full scintigraphic cohort was 8.5% (7/82); however, this figure warrants careful interpretation. Surgery was performed in only 30.5% of patients, and surgical indications, including large nodule size, compressive symptoms, or sonographic suspicion, inherently select for higher-risk cases. Indeed, the prevalence of overall malignancy or UMP among surgically treated patients was 60.0% (15/25), substantially exceeding rates reported in unselected thyroid surgery series and confirming that our surgical cohort was enriched for neoplastic disease. Accordingly, the 8.5% figure represents an observed frequency within a referral-based, surgically selected population rather than a true population-level prevalence of malignancy in all patients with scintigraphic evidence of toxic nodular disease. The more clinically transparent estimate may be the 28.0% rate among surgically treated patients (7/25; 95% CI, 14.1 to 47.8%), which reflects the diagnostic yield in patients who proceeded to thyroidectomy. Despite this caveat, comparison with prior literature remains informative. A systematic review by Mirfakhraee et al. and a recent multi-institutional surgical series by Koelliker et al. reported malignancy rates of approximately 3 to 4% and 7.3%, respectively, in resected solitary toxic nodules [9,20]. These figures were derived from similarly surgery-selected populations and therefore are subject to comparable selection pressures. The consistency across these series suggests that, within the subset of patients with hyperfunctioning nodules who ultimately undergo surgery, the risk of harboring a true hyperfunctioning neoplasm is not negligible and warrants lesion-level pathologic correlation.

Our findings also suggest that the pathologic spectrum of true hyperfunctioning neoplasms differs from that of coexisting cancers in the same gland. In our series, follicular-patterned diagnoses, including FT-UMP, OT-UMP, FTC, and FV-PTC, predominated among hyperfunctioning neoplasms, whereas coexisting tumors were usually small papillary carcinomas. This distinction is clinically relevant because aggregate cancer prevalence in toxic nodular disease does not distinguish cancers arising within the hyperfunctioning nodule from those occurring in separate nonfunctioning nodules, and therefore should not be directly interpreted as the malignancy risk of the hot nodule itself. Earlier studies have shown that thyroid cancers in hyperthyroid glands may arise either within the hot nodule itself or in separate nonfunctioning nodules [14]. Our results further suggest that these two scenarios may differ not only in location, but also in tumor size and histologic pattern.

Preoperative recognition of hyperfunctioning carcinoma or UMP remains challenging. In our cohort, these nodules frequently lacked classic high-risk sonographic features, and cytology was often nondiagnostic or indeterminate; one FV-PTC was interpreted as Bethesda II preoperatively. This pattern is compatible with the known limitation of cytology in follicular-patterned neoplasms, in which definitive diagnosis depends on histologic assessment for capsular or vascular invasion rather than cytomorphology alone [21,22]. In the present cohort, no ATA high-suspicion sonographic pattern was identified among the hyperfunctioning CA/UMP lesions, and ATA sonographic patterns did not significantly differ from those of the benign pathology group. Therefore, our findings do not support routine FNA of all clearly hyperfunctioning nodules or a size-based FNA threshold. However, a large or clinically dominant hyperfunctioning nodule, particularly when accompanied by compressive symptoms or indeterminate cytology, may warrant closer clinical assessment and consideration of definitive management. In such cases, FNA may provide additional information but should not be regarded as a reliable method for excluding follicular-patterned neoplasia. The inclusion of FT-UMP and OT-UMP also highlights that the spectrum of neoplasia in hyperfunctioning nodules may extend beyond overt carcinoma. Contemporary thyroid tumor classifications recognize low-risk follicular-patterned borderline tumors as distinct from unequivocal carcinoma [12,13]. In our cohort, no structural recurrence or distant metastasis was observed during available follow-up. These favorable outcomes should be interpreted cautiously in light of the small sample size and the inclusion of borderline malignancy such as FT-UMP [23].

Several limitations of this study must be acknowledged. First, this was a retrospective study conducted at a single tertiary center with a relatively small sample size, including only seven cases of hyperfunctioning carcinoma or UMP, which limits statistical power and generalizability. Second, there is an inherent selection bias, as the surgical cohort comprised only a fraction of patients with scintigraphic findings suggestive of toxic nodular disease. Because surgery was typically indicated for patients with large nodules, compressive symptoms, or clinical suspicion, the prevalence of neoplasia observed in this study may overestimate the risk in an unselected, medically managed hyperthyroid population. Third, precise attribution of pathologic findings to specific nodules in complex multinodular glands can be challenging, and despite careful preoperative mapping, some degree of misclassification cannot be entirely excluded. Finally, functional imaging in our cohort was predominantly performed using technetium-99m (Tc-99m) pertechnetate, which reflects tracer trapping but not iodine organification. As a result, some hyperfunctioning malignancies identified on Tc-99m scintigraphy could theoretically represent discordant nodules [24]. Even so, the tumor size difference between hyperfunctioning and coexisting tumors remained significant after sensitivity analysis excluding incidental microcarcinomas. In addition, a strength of this study is the integration of detailed lesion-specific pathology with a mean follow-up duration of approximately 50 months, providing longitudinal insight into the clinical course of these tumors. Furthermore, limited institutional follow-up data were available for a subset of the 57 patients who did not undergo surgery. Although no clinically diagnosed thyroid malignancy or newly documented high-suspicion sonographic features were identified during available follow-up, surveillance was incomplete and histopathologic confirmation was unavailable in the non-surgical cohort. Comprehensive long-term follow-up data would be invaluable for estimating true population-level malignancy rates in hyperfunctioning nodules. Prospective registry-based, multi-institutional studies with systematic pathologic correlation and next-generation sequencing are warranted to address this gap and to further elucidate the molecular basis of autonomous function in these neoplasms.

## 5. Conclusions

In conclusion, a hyperfunctioning thyroid nodule remains a strong negative predictor of malignancy, but hyperfunction does not definitively preclude carcinoma or UMP. The absolute frequency of malignancy in unselected hyperthyroid populations remains uncertain; the rates observed here reflect a surgically enriched cohort and should not be extrapolated to all patients managed with scintigraphy alone. The diagnostic significance of neoplasms in nodular hyperthyroidism depends on lesion attribution. In our cohort, definitive hyperfunctioning neoplasms were typically large, clinically dominant, and follicular-patterned, whereas coexisting cancers were usually small papillary tumors arising in nodules distinct from the hyperfunctioning nodule. These entities should therefore be interpreted separately when evaluating neoplasia in hyperthyroid patients with nodular disease.

## Figures and Tables

**Table 1:** Baseline clinical characteristics of the overall study cohort and comparison between the AFTN and TMNG subgroups.

Clinicopathological Feature	Total Cohort (*n* = 82)	Subgroup		
		AFTN (*n* = 45)	TMNG (*n* = 37)	*p*-Value
Age (years), mean ± SD	58.5 ± 14.6	59.7 ± 14.3	57.2 ± 15.0	0.441
Female, *n* (%)	63 (76.8%)	30 (66.7%)	33 (89.2%)	0.032 *
TSH (μIU/mL), median (range) ^a^	0.010 (0.008–0.400)	0.010 (0.008–0.591)	0.015 (0.008–0.400)	0.188
Free T4 (ng/dL), mean ± SD	1.45 ± 0.59	1.38 ± 0.55	1.55 ± 0.64	0.225
T3 (ng/dL), mean ± SD	128.8 ± 59.1	137.1 ± 69.9	119.9 ± 44.3	0.292
Surgery performed, *n* (%)	25 (30.5%)	17 (37.8%)	8 (21.6%)	0.150
Overall malignancy or UMP, *n* (%)	15 (18.3%)	11 (24.4%)	4 (10.8%)	0.153
Hyperfunctioning carcinoma or UMP, *n* (%)	7 (8.5%)	6 (13.3%)	1 (2.7%)	0.121
Hyperfunctioning carcinoma or UMP of surgical group ^b^, *n* (%)	7 (28.0%)	6 (35.3%)	1 (12.5%)	0.362

Notes: SD, standard deviation; TSH, thyroid-stimulating hormone; AFTN, autonomously functioning thyroid nodule; TMNG, toxic multinodular goiter; UMP, uncertain malignant potential. * *p* < 0.05. ^a^ TSH values below the detection limit (e.g., <0.008) were calculated as the limit value. ^b^ Proportion calculated within each subgroup. (AFTN = 17, TMNG = 8).

**Table 2:** Preoperative characteristics of the surgical cohort: hyperfunctioning carcinoma/UMP versus benign pathology group.

Clinicopathological Feature	Hyperfunctioning CA/UMP (*n* = 7) (4 Carcinomas; 3 UMPs)	Benign (*n* = 10)	*p*-Value
Age (years), mean ± SD	47.3 ± 17.6	54.9 ± 12.2	0.346
Female, *n* (%)	5 (71.4%)	9 (90.0%)	0.537
TSH (μIU/mL), median (range)	0.010 (0.008–0.040)	0.106 (0.008–0.345)	0.178
Free T4 (ng/dL), mean ± SD	1.2 ± 0.5	1.5 ± 0.7	0.258
Scan Impression, *n* (%)			0.338
AFTN	6 (85.7%)	6 (60.0%)	
TMNG	1 (14.3%)	4 (40.0%)	
Extent of surgery, *n* (%)			1.000
Lobectomy (Hemithyroidectomy)	2 (28.6%)	3 (30.0%)	
Total or Subtotal thyroidectomy	5 (71.4%)	7 (70.0%)	
ATA sonographic pattern, *n* (%)			0.593
Low suspicious	4 (57.1%)	8 (80.0%)	
Intermediate suspicious	3 (42.9%)	2 (20.0%)	
Preoperative FNA, *n* (%)			
Bethesda I (Non-diagnostic)	2 (28.6%)	2 (20.0%)	1.000
Bethesda II (Benign)	1 (14.3%)	3 (30.0%)	0.603
Bethesda III/IV (Indeterminate)	4 (57.1%)	2 (20.0%)	0.162
Not performed/Unknown	0 (0.0%)	3 (30.0%)	0.228

## Data Availability

The data presented in this study are available from the corresponding author upon reasonable request. The data are not publicly available due to privacy and ethical restrictions.

## Supplementary Materials

The following supporting information can be downloaded at: https://www.mdpi.com/article/10.3390/diagnostics16142249/s1, Table S1: Clinicopathologic Features of Seven Patients with Hyperfunctioning Thyroid Neoplasms; Table S2: (Nodule-Based). Pathological characteristics of malignant/UMP nodules: Hyperfunctioning vs. Coexisting (n = 18 nodules); Table S3: Pathological characteristics of malignant/UMP nodules: Hyperfunctioning vs. Coexisting tumors (≥1 cm).

## Abbreviations

The following abbreviations are used in this manuscript:

AFTN	Autonomously functioning thyroid nodule
ATA	American Thyroid Association
CA	Carcinoma
FNA	Fine-needle aspiration
FT-UMP	Follicular tumor of uncertain malignant potential
FTC	Follicular thyroid carcinoma
FV-PTC	Follicular variant papillary thyroid carcinoma
OT-UMP	Oncocytic thyroid tumor of uncertain malignant potential
I-131	Iodine-131
PTC	Papillary thyroid carcinoma
PTMC	Papillary thyroid microcarcinoma
Tc-99m	Technetium-99m
TMNG	Toxic multinodular goiter
TPOAb	Thyroid peroxidase antibody
TSHRAb	Thyroid-stimulating hormone receptor antibody
TSH	Thyroid-stimulating hormone
UMP	Uncertain malignant potential
