# Supplementary material for: Malignancy Risk Within Hyperfunctioning Thyroid Nodules: Clinicopathologic Features and Diagnostic Implications
Source: Diagnostics (Basel). 2026 Jul 18;16(14):2249. doi: 10.3390/diagnostics16142249 (PMC13409719; doi:10.3390/diagnostics16142249)
Supplement: Supplementary file 1 [file diagnostics-16-02249-s001.zip › diagnostics-4395592-supplementary.pdf]

Supplementary Table S1. Clinicopathologic Features of Seven Patients with Hyperfunctioning Thyroid Neoplasms

Case	Age / Sex	TSH	Scintigraphic Pattern	Sonographic pattern	FNA Cytology (Bethesda System)	Surgical Pathology	Tumor Size (cm)	Pathologic Stage
**1**	24 / F	<0.008	Toxic Adenoma (Rt)	Low suspicious	I (Non-diagnostic)	FTC	5.5	pT3N0M0
**2**	57 / M	0.008	Toxic Adenoma (Rt)	Low suspicious	IV (Follicular Neoplasm)	FTC	4.5	pT3aNx
						Coexisting PTMC (Lt)		pT1aNx
**3**	67 / F	0.01	TMNG (Mixed pattern)[ˆ]	Intermediate suspicious	III (AUS)	OT-UMP	8.5	N/A
						ectopic PTMC [Δ]		pT1a(PTMC)
**4**	48 / M	0.021	Toxic Adenoma (Rt)	Intermediate suspicious	II (Benign)	FV-PTC	3	pT2N0
**5**	68 / F	0.01	Toxic Adenoma (Rt)[†]	Low suspicious	I (Non-diagnostic)	FT-UMP	8.6	N/A
**6**	37 / F	<0.01	Toxic Adenoma (Lt)	Low suspicious	IV (Follicular Neoplasm)	FT-UMP	3.5	N/A
**7**	30 / F	0.04	Toxic Adenoma (Lt)[†]	Intermediate suspicious	III (AUS)	PTC	1.3	pT1bN0a

F, female; **M**, male; **Rt**, right lobe; **Lt**, left lobe; **TMNG**, toxic multinodular goiter; **AUS**, atypia of undetermined significance; **FTC**, follicular thyroid carcinoma; **PTC**, papillary thyroid carcinoma; **FV-PTC**, follicular variant of papillary thyroid carcinoma; **FT-UMP**, follicular tumor of uncertain malignant potential; **OT-UMP**, Oncocytic (Hürthle cell) thyroid tumor of uncertain malignant potential.

Notes: ˆ Scintigraphy showed multiple nodules bilaterally with a dominant hyperfunctioning focus in the left lobe corresponding to the OT-UMP. Δ An incidental ectopic papillary microcarcinoma (0.3 cm) was found in the central neck. † Scintigraphy was performed with I-131 NaI.

Supplementary Table S2. (Nodule-Based). Pathological characteristics of malignant/UMP nodules: Hyperfunctioning vs. Coexisting (n = 18 nodules)

Characteristic	Hyperfunctioning CA/UMP Nodules (n = 7)	Coexisting Tumor (n = 11)	P-value
**Tumor origin**	Hyperfunctioning lesion itself	Collision or non-functioning	-
**Max Tumor Size (cm), mean ± SD**	5.0 ± 2.8	0.8 ± 0.7	0.007
**Primary pathology type, n (%)**			
**Follicular patterned (FTC/UMP/FV-PTC)**	6 (85.7%)	3 (27.3%)	0.0498
**Classic/Micro Papillary (PTC/PTMC)**	1 (14.3%)	8 (72.7%)	-

Notes: P values were calculated using Fisher's exact test for categorical variables and Welch's t-test for continuous variables. $P < 0.05$ was considered statistically significant.

Supplementary Table S3. Pathological characteristics of malignant/UMP nodules: Hyperfunctioning vs. Coexisting tumors (≥ 1cm)

Characteristic	Hyperfunctioning CA/UMP Nodules (n = 7)	Coexisting Tumor ≥ 1cm (n = 6)	P-value
**Max Tumor Size (cm), mean ± SD**	5.0 ± 2.8	1.4 ± 0.3	0.014
**Primary pathology type, n (%)**			0.266
**Follicular patterned**	6 (85.7%)	3 (50.0%)	
**Classic Papillary**	1 (14.3%)	3 (50.0%)	
